# Huge mucinous cystic neoplasms with adhesion to the left colon: A case report and literature review

**DOI:** 10.1515/med-2022-0612

**Published:** 2022-12-31

**Authors:** Haijun Tang, Zhihong Shen, Baochun Lu

**Affiliations:** Hepatopancreatobiliary Surgery Department, Shaoxing People’s Hospital, Shaoxing, Zhejiang, 312000, China; Hepatopancreatobiliary Surgery Department, Shaoxing People’s Hospital, Shaoxing, Zhejiang, 312000, China

**Keywords:** mucinous cystic neoplasms, precancerous conditions, tissue adhesions, colon

## Abstract

Mucinous cystic neoplasms (MCN) are rare premalignant neoplasms of the pancreas typically found as single lesions in the pancreatic body and tail of women in the fifth and sixth decade of life, constituting 2–5% of pancreatic neoplasms. We present a 50-year-old female patient with a large tail mass of the pancreatic body (size of 15 cm × 12 cm) with elevated tumor indicators. Computed tomography and magnetic resonance imaging revealed a large cystic lesion with septa and wall nodules. During the operation, the tumor capsule wall adhered to the left half colon and mesentery and could not be detached. Splenectomy and left hemicolectomy were performed. The postoperative pathological examination of the specimens confirmed a premalignant pancreatic mucous cystic tumor with moderate heterocytosis. The preoperative diagnosis of pancreatic MCN and MCN with invasive carcinoma is discussed, considering the characteristics of this case. Age, tumor size, texture, tumor marker elevation, and cystic wall condition are important characteristics of malignant MCN. Nevertheless, it is still very difficult to determine accurately whether an MCN is malignant or not before an actual pathological examination of the resected specimen.

## 1 Introduction

Mucinous cystic neoplasms (MCN) are rare premalignant neoplasms of the pancreas typically found as single lesions in the pancreatic body and tail of women in their fifth and sixth decade of life, representing 2–5% of all pancreatic neoplasms [1–3]. MCN has a clear tendency to be malignant and is never truly benign [2–5]. MCN progresses slowly, with invasive carcinoma ultimately found in 6–36% of patients with MCN [2–5]. Although age, tumor size, texture, tumor marker elevation, and cyst wall condition are considered as important characteristics of malignant MCN, it is still very difficult to determine whether an MCN is malignant or not preoperatively [1–6]. Therefore, surgery is required to determine the exact nature of the MCN lesion [1–6].

Here, we report a middle-aged female with a large 15 cm × 12 cm MCN with the capsule adhering to the left half colon and mesentery. Before and after the operation, it looked more like an invasive MCN, and distal pancreatectomy, splenectomy, and left hemicolectomy were performed.

## 2 Case report

A 50-year-old female patient was hospitalized on December 12, 2019, due to back pain for 2 years. She had no history of trauma or other diseases. On abdominal examination, a 10 cm × 10 cm mass in the left abdomen was found, with a clear boundary and poor mobility. The abdomen was soft, and there was no tenderness. The other physical examinations were unremarkable.

**Figure 1:**
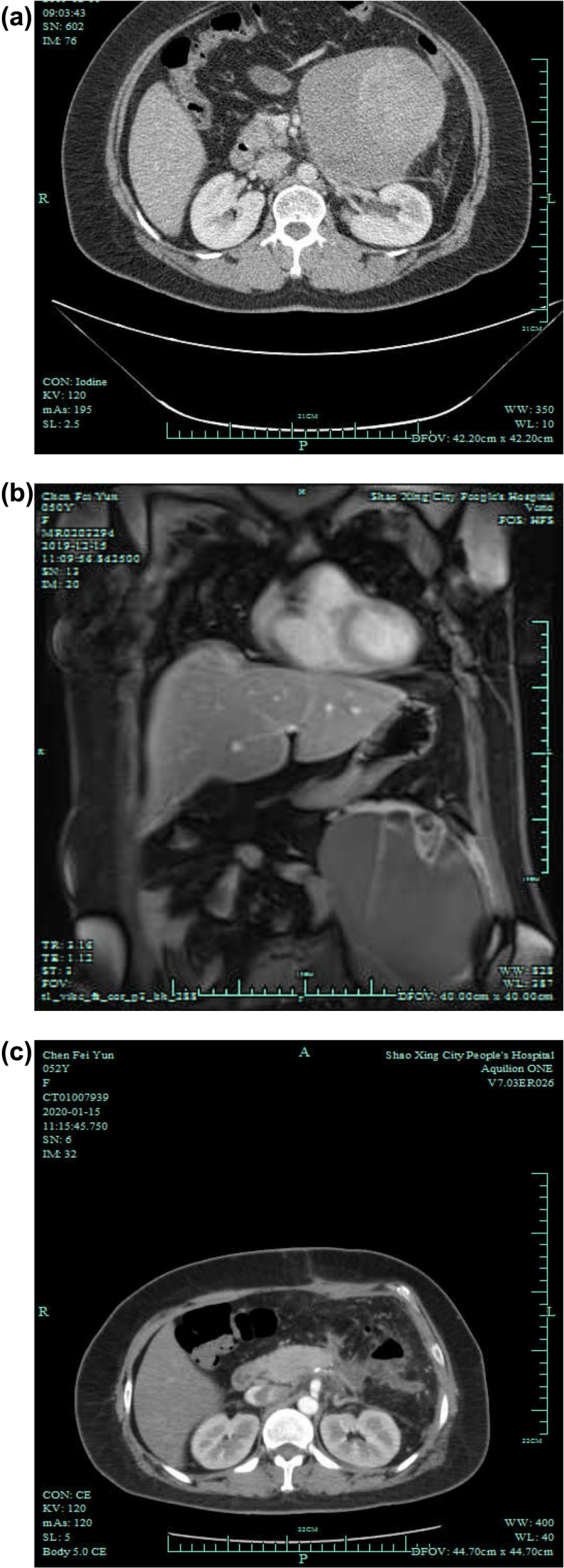
Imaging examinations. (a) Preoperative CT. (b) Preoperative MRI. The left abdominal cavity shows a massive mass (15 cm × 12 cm). There were septa and wall nodules, and the enhanced scanning septum and wall nodules were significantly enhanced. The adjacent peritoneum was slightly thickened, and the adjacent pancreas was pressurized and pushed upward, with an unclear boundary with the lesion. (c) Postoperative CT at 3 months after surgery.

Table 1 presents the tumor marker levels at admission. Abdominal enhanced computed tomography (CT) revealed a large mass with an unclear boundary at the lower margin of the pancreas (Figure 1a). Abdominal enhanced magnetic resonance imaging (MRI) revealed a large mass with slightly high T1 and T2 signals in the left abdominal cavity, with a size of 15 cm × 12 cm. There were septa and wall nodules (Figure 1b). There was a patch of short T1 signal at the wall nodules, and the scanning interval and wall nodules were significantly enhanced. The adjacent pancreas was compressed and pushed upward, with unclear lesions’ boundaries. The tumor was in the left abdomen, in the body and tail of the pancreas, posterior to the stomach and adjacent to the spleen. No enlarged lymph nodes and mass shadows were observed in the retroperitoneum. The abdominal wall had no special structure (Figure 1). No obvious abnormality was found in blood, stool, hepatic and renal functions, and coagulation function tests.

**Table 1:** Tumor marker levels at admission

Marker	Unit	Value	Reference value
CA242	IU/mL	122.3	<20.0
CA50	IU/mL	>500	<25.0
CA724	IU/mL	14.33	<6.9
CEA	ng/mL	10.51	<3.4
CA199	U/mL	3379.84	<35
CA125	U/mL	47.6	<39

**Figure 2:**
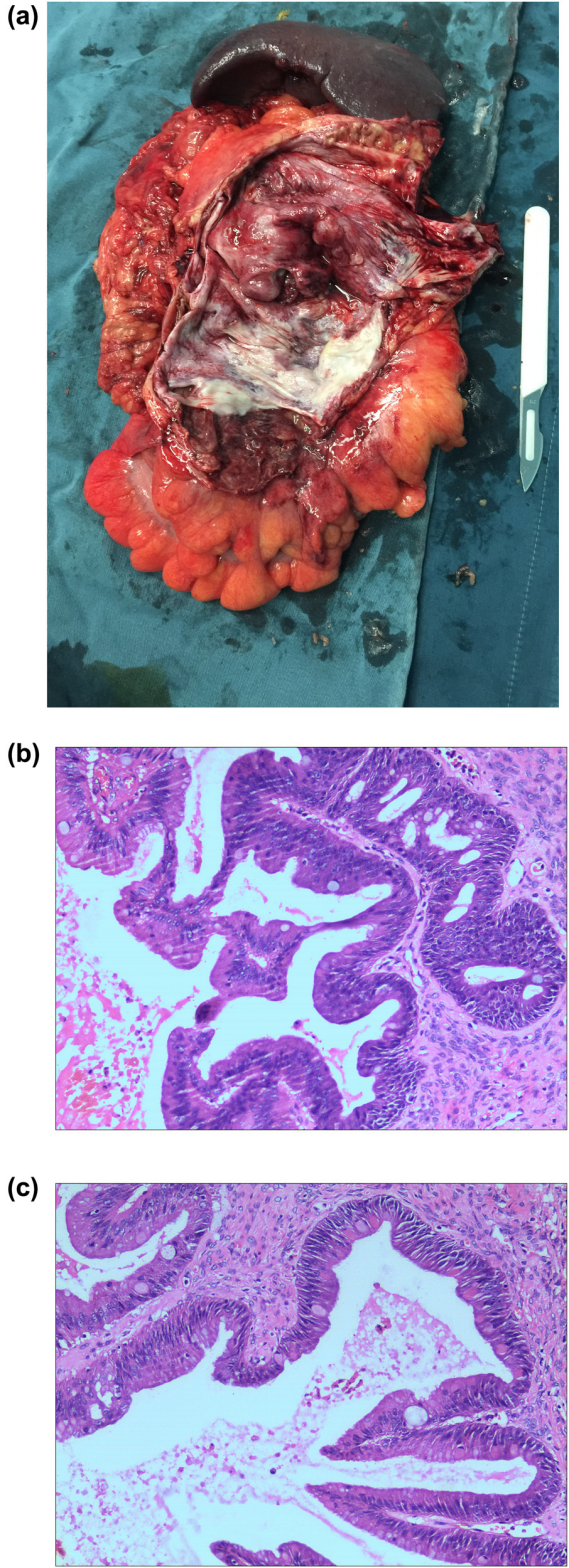
(a) Gross examination showing a 27 cm long, 2.5 cm diameter colon specimen without a difference in the mucous membrane. The external side of the intestinal wall showed a cystic neoplasm of 16 cm × 12 cm × 3.5 cm, with a cystic wall thickness of 0.3–1.4 cm. The multilocular lesion contained a turbid purulent thick liquid. Most of the capsule wall was smooth, with local, visible nodular protrusions. The cystic wall originated near the pancreas, which was 12 cm × 3.5 cm × 1.4 cm. The spleen was 14 cm × 8 cm × 2.5 cm, palm red, soft, and without obvious nodules and masses. (b) Well-differentiated columnar cells and ovarioid stroma (×20). (c) Cells were arranged in pseudostratification. The nuclei were enlarged and crowded, vacuolated, and visible (×400).

The preoperative diagnosis was MCN, but malignant changes could not be excluded. An exploratory laparotomy was performed on December 19, 2019, and a large thick-walled cystic lesion filled with a turbid purulent thick liquid was found (Figure 2a). The upper boundary of the tumor was intimately adhering to the pancreatic tail, with obvious infiltration, and the tumor capsule wall also had dense adhesions to the left half colon and mesentery and could not be detached. The intraoperative pathological examination suggested an MCN with moderate hyperplasia, and malignancy could still not be excluded. Therefore, the operation was expanded, and distal pancreatectomy, splenectomy, and left hemicolectomy were performed.

The postoperative pathology suggested that the tumor was multilocular and cystic. The tumor was composed of two distinct components: epithelial cells and dense spindle cells (Figure 2b). At high magnification, a small number of tumor cells were well-differentiated and columnar (Figure 2c). The nuclei were in the cell basement, and there was no atypia. There were mucous vacuoles in the cytoplasm. The glands were disorganized, and part of the epithelium formed a papillary structure. The cells were pseudostratified. The nuclei were enlarged, crowded, vacuous, and visible (Figure 2c). A mitotic figure could be seen.

A pancreatic fistula occurred 1 week after surgery, with about 30 mL of drainage fluid daily. The drainage fluid was turbid with elevated amylase (19,610 U/L). No drainage tube blockage or abdominal pain was observed. The amount of drainage fluid decreased after 3 weeks without treatment. The follow-up showed no abnormality by June 2020. A follow-up CT scan revealed no obvious lesion (Figure 1c).


**Ethics approval and patient consent:** The study was approved by the Ethics Committee of Shaoxing People’s Hospital. Written informed consent has been obtained from the patient in this study.

## 3 Discussion

In 2010, the World Health Organization divided MCNs into three subtypes: MCN with low- or moderate-grade atypia, MCN with high-grade dysplasia, and MCN with invasive carcinoma [7]. MCN is a rare occurrence, but with the development of imaging techniques, the incidence of MCN has increased significantly [1,8]. MCN is a cyst-forming tumor produced by columnar epithelial cells, with ovarian stroma and potential malignancy, especially in patients with a long disease course and large tumors [7]. MCN and MCN with invasive carcinoma are difficult to distinguish clinically. The clinicopathological features and imaging findings of pancreatic MCN malignancy have been reported in different studies [2].

Yamao et al. [3] showed that age and tumor size were predictors of the benign/malignant nature of the lesion. They showed that 56 years of age and a tumor size of 51 mm were the thresholds suggesting malignancy, while sex, symptoms, and tumor location were not associated with malignant MCNs.

Most laboratory examinations of patients with MCN are within the normal ranges, but most patients with MCN and invasive carcinoma show CA199 elevation, and the literature suggests that elevation of carcinoma embryonic antigen (CEA) or CA199 indicates an increased possibility of malignancy [9]. Endoscopic ultrasound (EUS) is an ideal diagnostic method for a pancreatic tumor. In addition to obtaining high-resolution images of the lesion, it can also be used for biopsy. When EUS is combined with CEA and CA199 to diagnose cystadenocarcinoma, the sensitivity reaches 94.4% [10,11]. B-mode ultrasound or CT-guided percutaneous aspiration of vesicle fluid with positive detection of k-ras gene mutation is also suggestive of cystadenocarcinoma [10,12].

B-mode ultrasound examination can accurately locate the tumor, but the disadvantage is that the gastrointestinal tract is prone to gas interference. CT is a commonly used imaging examination method and has important value in the differential diagnosis of pancreatic mucinous cystic tumors. It can detect cystic pancreatic lesions and show the imaging characteristics of the cystic wall and lumen. Visser et al. [13] reported that in all kinds of imaging examinations, the diagnostic accuracy of MRI and CT ranged from 44 to 83%, and the imaging performance of MRI cross-section was roughly the same as that of CT. Another study showed that MRI was superior to CT for the diagnosis of MCN (45 vs 9%) [6]. Yan et al. [14] reported that the thickness of the capsule wall and the presence of solid components could be important differentiating factors between benign and malignant pancreatic MCNs, as supported by the case reported here. The differences in the thickness of the capsule wall, the heterogeneous enhancement of the capsule wall, and the heterogeneous enhancement of the solid component between the benign and the malignant groups were statistically significant.

The preoperative examinations can provide some reference for the preoperative prediction of pancreatic mucous cystadenocarcinoma and pancreatic mucous cystadenoma. Nevertheless, the diagnosis still requires pathological confirmation. Benign and malignant epithelial cells can coexist in MCNs and must be removed. A simple biopsy is unreliable since it can easily miss small malignant foci. Only a careful examination of the specimen can confirm a benign diagnosis; otherwise, there is the possibility of a misdiagnosis of cystadenocarcinoma [15]. In the case reported here, several preoperative aspects (tumor size, tumor index elevation, imaging features, and the intraoperative relationship between the tumor and surrounding tissues) suggested mucinous cystadenocarcinoma, but pathologically, it was confirmed as mucinous cystadenoma with moderate heterocytosis, which is a precancerous lesion.

Since MCN has a malignant tendency and is not sensitive to chemotherapy or radiotherapy, surgery is the preferred treatment [4]. MCN has a good prognosis, and the specific surgical method should be determined according to the tumor location, size, relationship with surrounding tissues, and intraoperative freezing pathology. Most patients with invasive MCN cancer can also be cured by surgery [5].

A literature review was performed in PubMed using the keywords (“mucinous cystic neoplasm”) AND (case report) AND (pancreatic) AND (big or huge or large). Only the articles published from 2000 to now were kept. Cases with tumors <10 cm, not MCN, and published in another language than English were excluded. Finally, 15 articles were included [16–30]. Table 2 presents the 15 cases. These 15 cases are relatively heterogeneous, and it is difficult to draw a relationship between imaging and malignancy from them. Still, the cases with malignant lesions at final pathological examination usually showed complex cystic masses with intracystic masses, necrosis, and hemorrhage. Still, complex cystic masses were also observed in some benign cases. Of course, considering the size of the lesions, invasive foci might have been missed at microscopic examination.

**Table 2:** Summary of case reports of MCN

Reference	Year	Age (year)	Tumor size (cm)	Serum tumor marker	Imaging features	Intraoperative relationship between the tumor and surrounding tissues	Nature of MCN
Carvalho [16]	2020	32	24 × 17 × 13	CEA, CA19-9, CA15-3, and CA125 were negative	Complex cystic lesion involving the tail of the pancreas had two solid components. A few septa inside the cystic mass.	Smooth tumor surface with no communication with the pancreatic ductal system.	Low-grade dysplasia, non-invasive.
Paniccia [18]	2017	36	10 × 8	Normal CEA, elevated CA19	Thick walled with multiple internal septa and both cystic and solid components.	The pancreatic margin was normal pancreas parenchyma with low-grade PanIN.	High-grade dysplasia. Microscopic invasive well-differentiated adenocarcinoma. Areas of undifferentiated malignancy with sarcomatous features.
Munekage [20]	2016	25	10 × 12.5	Normal CA19-9, elevated CA125, and PCAA2	Large complex cystic and solid mass with a thick capsule. Disruption of the major pancreatic duct.	Smooth external surface. The mass was filled with mucinous fluid and had a solid component.	Invasive mucinous cystadenocarcinoma with anaplastic carcinoma.
Tica [24]	2013	27, pregnant	11.6 × 10.3 × 10.5	CEA, CA19-9, CA15-3, and CA125 were normal	Ultrasound showed a large multilocular hypoechoic cystic tumor, having a thick hyperechogenic external wall, with no papillary projections insides, but with vascularity present in some septa. MRI showed a multilocular cystic mass in the body and tail of the pancreas.	Smooth tumor surface with no communication with the pancreatic ductal system.	Benign pancreatic mucinous neoplasms, with tumor-free margins and non-affected lymph nodes.
Tsuda [17]	2012	28	15 × 14	CA19-9 was normal, CA125 was high (76.3 U/mL)	Mural nodules and hypertrophic septa partially with the presence of blood flow inside the tumor. MRI showed a multiloculated cystic tumor.	Smooth cystic tumor arising from the body and tail of the pancreas and adhering to the spleen and retroperitoneum.	Premalignant condition.
Ghatak [26]	2012	35	37 × 25 × 8	—	Complex hepatic mass having cystic, solid, and fatty areas and a similar complex mass inside the abdominal cavity.	Partly cystic and partly fatty mass, arising from the head of the pancreas. Abundant fat around the portal triad and beneath the gallbladder.	Mucinous neoplasm with a single layer of mucin secreting columnar epithelium. No cellular stratification, pleomorphism, or mitotic activity.
Naganuma [19]	2011	32, pregnant	11 × 9 × 5	Normal CEA, elevated CA19	Ultrasound showed a honeycomb-like cystic lesion in the right upper abdomen. CT revealed a multilocular cystic lesion with hemorrhage in the right upper quadrant of the abdomen.	Ruptured mass in the right anterior portion and copious mucinous fluid containing necrotic material in the abdominal cavity.	Mucinous cystic neoplasm with adenocarcinoma.
Nakamura [29]	2011	37	17	—	Cystic tumor occupying almost the entire distal portion of the pancreas and the spleen was enlarged. No nodular lesion was observed in the cyst.	The pancreatic neck had not been infiltrated by the tumor. The distal pancreas was occupied by the tumor.	Mucinous cystic neoplasm.
Mizutani [27]	2009	29	15 × 15	Normal CA19-9, PMAT2, and CEA	Ultrasound showed a hypoechoic cystic tumor in the body and tail of the pancreas. Dynamic CT and MRI revealed a 15 cm-diameter cystic tumor with many septa and a thin cystic wall with partial calcification.	Patterned, indented surface with a thick capsule and no invasion of surrounding structures was recognized.	Mucinous cystic adenocarcinoma *in situ*.
Hisa [28]	2009	60	24	—	Ultrasound showed an oval cystic mass filled with debris. CT showed internal vessels without peripheral enhancement, and the cyst wall was thin.	A deviation of the main pancreatic duct without mucin or communication with the cyst.	Mucinous cystadenoma.
Ikuta [22]	2008	30, pregnant	18 × 14	—	Well-demarcated cystic mass originating from the distal pancreas. No septa or protruding lesions inside the cystic mass.	Unilocular mass with thick walls and filled with tenacious mucoid material and necrotic debris.	Mucinous cystadenocarcinoma with moderate dysplasia.
Ishigami [21]	2007	35	15	Elevated CA19-9	Large lobulated cystic tumor at the pancreatic tail.	Round tumor in the upper left abdomen. No invasion of neighboring organs or the portal vein was apparent.	Mucinous cystadenocarcinoma with no invasive component.
Ishikawa [23]	2007	33, pregnant	12	—	Ultrasound showed a large multilocular hypoechoic mass with septa in the left upper abdomen. MRI showed a multilocular cystic lesion without any solid components in the body and tail of the pancreas.	Huge, smooth cystic tumor was found arising from the body and tail of the pancreas and adhering to the spleen, mesocolon, and retroperitoneum.	Benign mucinous cystadenoma with tumor-free tissue margins.
Kitagawa [30]	2006	25, pregnant	15 × 15	Increased level of CA19-9 (3,090 U/mL)	Abdominal CT and upper abdominal ultrasound showed a large cystic mass in the body of the pancreas.	A normal main pancreatic duct and no detectable connection between this duct and the tumor.	Mucinous cystic adenoma of the pancreas.
Lopez-Tomassetti Fernandez [25]	2005	26, pregnant	14 × 10 × 14	—	Ultrasound demonstrated a well-delimited cystic mass in the left upper abdominal quadrant.	A yellowish well-delimited cystic mass with a smooth surface, with no evidence of invasion.	Mucinous cystic neoplasm of pancreas considered to be a premalignant lesion.

MCN: mucinous cystic neoplasms; CEA: carcinoma embryonic antigen; CA: carbohydrate antigen; MRI: magnetic resonance imaging; CT: computed tomography.

With the improvements in imaging technologies and the use of screening CT and MRI, the incidence of MCN is expected to increase, and clinicians must be aware of its possibility and management. In addition to the traditional and already known risk factors for MCN (i.e., women in their fifth and sixth decade of life), this case report and literature review suggest that tumor size, elevated tumor markers, tumor texture, and cystic wall at imaging might be indicative of a higher likelihood of malignant MCN. Nevertheless, since the lesion’s final benign or malignant nature can only be determined pathologically, the best course of action remains surgery, and the patients should be referred accordingly. A multidisciplinary team discussion (including radiologists, surgeons, pathologists, oncologists, and radiation oncologists) is also probably warranted to explore all treatment options.

In conclusion, age, tumor size, texture, tumor marker elevation, and cystic wall condition are important characteristics of malignant MCN. Nevertheless, it is still very difficult to accurately determine whether the exact nature of MCN is malignant or not before an actual pathological examination of the resected specimen.
